# Antitumor effects of metformin via indirect inhibition of protein phosphatase 2A in patients with endometrial cancer

**DOI:** 10.1371/journal.pone.0192759

**Published:** 2018-02-14

**Authors:** Shinsuke Hanawa, Akira Mitsuhashi, Makio Shozu

**Affiliations:** Department of Reproductive Medicine, Graduate School of Medicine, Chiba University, Chiba, Japan; Sackler School of Medicine, Tel Aviv University, ISRAEL

## Abstract

**Objective:**

Metformin, an antidiabetic drug, inhibits the endometrial cancer cell growth in vivo by improving the insulin resistance; however, its mechanism of action is not completely understood. Protein phosphatase 2A (PP2A) is a serine/threonine phosphatase associated with insulin resistance and type 2 diabetes, and its inhibition restores the insulin resistance. This study investigated the antitumor effect of metformin on endometrial cancer with a focus on PP2A.

**Methods:**

Metformin (1,500–2,250 mg/day) was preoperatively administered to patients with endometrial cancer for 4 to 6 weeks. Expression of the PP2A regulatory subunits, 4 (PPP2R4) and B (PP2A-B), was evaluated using real-time polymerase chain reaction (RT–PCR) and immunohistochemistry (IHC) using paired specimens obtained before and after metformin treatment. The effect of *PPP2R4* inhibition with small interfering RNA was evaluated in the endometrial cancer cell lines HEC265 and HEC1B. P values of < .05 were considered statistically significant.

**Results:**

Preoperative metformin treatment significantly reduced the expression of PP2A-B, as determined using IHC, and the mRNA expression of *PPP2R4*, as determined using RT–PCR, in the patients with endometrial cancer. However, metformin could not directly alter the *PPP2R4* mRNA levels in the endometrial cancer cell lines in vitro. *PPP2R4* knockdown reduced the proliferation and induced the apoptosis by activating caspases 3/7 in HEC265 and HEC1B cells.

**Conclusions:**

Downregulation of the PP2A-B subunit, including PPP2R4, is an important indirect target of metformin. Inhibition of PP2A may be an option for the treatment of endometrial cancer patients with insulin resistance.

**Trial registration:**

This trial is registered with UMIN-CTR (number UMIN000004852).

## Introduction

Endometrial cancer (EC) is the most common gynecological malignancy and the fourth most common cancer in US women[1]. Among the various cancers, EC has the strongest association with obesity [2, 3]. Insulin resistance and type 2 diabetes mellitus caused by obesity are recognized as risk factors for endometrial cancer [4, 5]. Improvement of insulin resistance and abnormal glucose metabolism has been considered a preventive and therapeutic target.

Metformin, an oral biguanide antihyperglycemic drug, is widely prescribed as a first-line therapy against type 2 diabetes mellitus [6]. Besides its effectiveness in diabetes treatment, several population studies have identified additional benefits of metformin, including the metformin-induced decrease in cancer incidence and cancer-related mortality in patients with diabetes [7]. In addition, many *in vitro* studies have shown that metformin exerts antineoplastic effects on various types of cancer cells [8]. However, the metformin concentration used in *in vitro* studies was much higher than the established *in vivo* concentration of orally administered metformin, thus raising questions as to whether such *in vitro* antitumor effects are clinically relevant [8]. In this regard, window of opportunity studies have revealed that the antidiabetic dose of metformin causes growth inhibition in breast cancer and EC *in vivo* [9–12]. This effect is likely because of an indirect alteration of an endocrine metabolic factor; however, the precise mechanism of the anticancer efficacy of metformin has not been elucidated yet. We have previously reported that preoperative metformin treatment significantly reduced the expression of the Ki-67 protein and topoisomerase IIα in EC [10]. This result has been supported by several other reports [11, 12] confirming that metformin reduces the tumor proliferation in type 1 EC tissues. However, the mechanism is not clearly understood.

Protein phosphatase 2A (PP2A) is a major serine/threonine phosphatase found in cells and possessing diverse functions. Activation of PP2A is associated with insulin resistance and type 2 diabetes [13, 14], whereas inhibition of PP2A results in enhanced glucose homeostasis and increased insulin sensitivity [15]. PP2A has also been known as a tumor suppressor[16]. Okadaic acid, a potent inhibitor of PP2A, and several endogenous PP2A inhibitors such as CIP2A and SET have been shown to promote the malignant growth of human cancers [17].

In contrast, some studies have shown that PP2A may have an oncogenic role [17–19]. Overexpression of PP2Ac in hepatocellular cancer models inhibits p53-mediated apoptosis [18]. Some mutations, including *PPP2R1A* mutation, may be gain-of-function rather than loss-of-function mutation [20]. *PPP2R1A* mutation has been observed at high frequency in endometrial serous carcinoma [21] and it promotes cancer cell growth [20]. Overexpression of wildtype PPP2R1A increased cell proliferation *in vitro* and tumor growth *in vivo* in endometrial serous carcinoma [22]. Inhibition of PP2A has been considered a therapeutic target in these cancers. Additionally, PP2A inhibitors, such as cantharidin and norcatharidin, have been found to repress the invasion of cancer cells and induce apoptosis of cancer cells [17, 23, 24]. LB-100, a small-molecule inhibitor of PP2A, sensitizes ovarian cancer cells to cisplatin *in vitro* and *in vivo* [25].

In the present study, we investigated the antitumor effect of metformin and its relationship with PP2A in patients with EC. An antidiabetic therapeutic dose of metformin was found to indirectly inhibit the EC cell growth *in vivo* and reduce the PP2A expression. Furthermore, we focused on the PP2A regulatory subunit 4 (PPP2R4), which is required for PP2A regulation [26–28]. We showed that the inhibition of PPP2R4 reduced the proliferation ability of EC cells and increased the activity of caspases.

## Patients and methods

### Patients

Twenty seven patients with endometrioid carcinoma, who were treated with metformin preoperatively, were included in this study. All the patients were recruited for our previous study between January 2011 and May 2013, which was registered with the University Hospital Medical Information Network (UMIN) Clinical Trial Registry (UMIN 000004852), and provided re-consent for this study.

The eligibility criteria were those with an in the Eastern Cooperative Oncology Group performance status of 0 to 1, as well as normal renal, liver, and cardiac function. The exclusion criteria were as follows: 1) type 2 diabetes requiring medication; 2) a history of metformin use; 3) an abnormal blood coagulation profile and/or a history of thromboembolism; and 4) the presence of mental or life-threatening illnesses.

Immunohistochemistry (IHC) was performed on samples from 27 patients, and RNA was isolated from samples collected from six patients. As an IHC control, EC specimens embedded in paraffin blocks were retrospectively collected from 10 patients with endometrioid carcinoma, who underwent surgery at the Chiba University Hospital but did not receive metformin. We also used the normal endometria of six women who agreed to participate as a control for *PPP2R4* mRNA expression in this study. These women with normal endometria had no medical history of EC and no obesity.

### Study design

The primary objective of the present study was to evaluate the relationship between PP2A regulation and the growth inhibitory effect of metformin in EC tissues. The secondary objective was to evaluate the effect of PPP2R4 inhibition on EC cells *in vitro*. Metformin (initial dose, 750 mg/day; increased weekly up to 1,500 or 2,250 mg/day) was administered for 4–6 weeks until the day of scheduled surgery. Tissue specimens were obtained via endometrial curettage at the time of initial diagnosis (before treatment) and hysterectomy (after treatment). Changes in PP2A expression were determined using IHC and real-time polymerase chain reaction (PCR) using paired endometrial tissue specimens.

The Institutional Review Board of Chiba University approved the study protocol, and all patients provided written informed consent before participation. S1 File. shows the Transparent Reporting of Evaluations with Nonrandomized Designs (TREND) checklist of the study.

### Cell lines, cell culture and reagents

Type 1 EC model cell lines, HEC265 and HEC1B, were cultured in Dulbecco's modified Eagle’s medium (Gibco, Life Technologies Corporation, Carlsbad, CA, USA) containing 4.5 g/L glucose, 10% fetal bovine serum (FBS; Sigma–Aldrich, St. Louis, MO, USA), 100 U/mL penicillin, and 100 μg/mL streptomycin at 37°C in an atmosphere of 5% CO_2_. The HEC265 and HEC1B cell lines were purchased from the JCRB Cell Bank (Osaka, Japan). Antibodies for PP2A subunit B (PP2A-B; #4953), phosphotyrosyl phosphatase activator/PPP2R4 (#3330), phospho-p44/42 mitogen-activated protein kinase (MAPK) [extracellular signal-regulated kinase (ERK1/2), Thr202/Tyr204; #4370], p44/42 MAPK (ERK1/2; #9102), cyclin D1 (#2922), phosphor-ribosomal protein S6 (phosphor rpS6) (Thr389; #2215), rpS6 (#2217), phospho-glycogen synthase kinase-3 beta (GSK3β)(Ser9; #5558), GSK3β (#9315), phospho-protein kinase B (AKT, Thr308; #2965), AKT (#4691), and β-actin (#4967) were all purchased from Cell Signaling Technology, Inc. (Danvers, MA, USA). PPP2R4-targeting SMART pool small interfering RNA (siRNA) (siPPP2R4) and non-targeting siRNA (siControl) were purchased from Dharmacon (Lafayette, CO, USA). Metformin was obtained from Sigma–Aldrich Chemistry (Germany).

### Immunohistochemical analysis

PP2A in EC tissues was evaluated using IHC staining of PP2A-B. Tissue sections (3-μm thick) were briefly microwaved in 10 mM citrate buffer (pH 6.0) and then immunostained for PP2A-B. The EnVision FLEX mini kit (K8000; Dako Denmark A/S) and an autostainer S3400 (Dako Denmark A/S) were used to visualize immunostaining. The tissue sections were incubated with the primary antibody (dilution 1:100) at room temperature for 60 min. Then, the secondary antibody [EnVision FLEX/horseradish peroxidase (HRP); Dako Denmark A/S] was added, followed by incubation at room temperature for 60 min, and 3,3’-diaminobenzidine tetrahydrochloride (Dako Denmark A/S) was used as a chromogen. The samples were counterstained with hematoxylin. The stained samples were observed and graded semiquantitatively as negative, weakly positive, moderately positive, and highly positive.

Paired specimens obtained at the time of preoperative biopsy and at surgery were stained for PP2A-B. Expression of PP2A was compared between pre- and postoperative tissues with or without metformin administration. The results of PP2A-B IHC were evaluated using immunoreactivity scores (IRSs) [29, 30] calculated as follows: IRS = staining intensity × percentage of positive cells. The staining intensity was categorized as follows: 0 (negative), 1 (weak), 2 (moderate), and 3 (strong). Positive cells were categorized as follows: 0 (negative), 1 (< 10%), 2 (10–50%), 3 (50–80%), and 4 (80–100%). The maximum IRS = 3 × 4 = 12.

### RNA isolation, reverse transcription, and real-time PCR

Total RNA was extracted from cells using the RNeasy mini kit (Qiagen, Germany). RNA samples were obtained from six patients because only six paired samples were evaluated. RNA concentrations were determined using a NanoDrop 1000 (Thermo Fisher Scientific, USA). cDNA was synthesized using the Superscript VILO cDNA synthesis kit (Invitrogen, USA) according to the manufacturer’s protocol. Absolute transcript levels were quantified using Light Cycler FastStart DNA Master SYBR Green I (Roche, USA) in a Lightcycler 2.0 (Roche, USA). The sense and antisense PCR primers used were as follows: for *PPP2R4*, 5’-AGGGTCTCATCCGCATGTAT- 3’ and 5’-CAGGCTCCCGAACTTGAA- 3’, respectively; and for β-actin, 5’-CCAACCGCGAGAAGATGA-3’ and 5’-TCCATCACGATGCCAGTG-3’, respectively. PCR was performed by initial denaturation at 95°C for 10 min, followed by 35 cycles of 10 s at 95°C, 10 s at 60°C, and 5 s at 72°C for β-actin or 35 cycles of 10 s at 95°C, 10 s at 59°C, and 4 s at 72°C for *PPP2R4*. The expression level of *PPP2R4* was normalized relative to that of β-actin. The relative quantitative value was obtained using the 2^−ΔΔCt^ method [31].

### siRNA transfection

The endometrial carcinoma cell lines were transfected with siPPP2R4 or siControl by reverse transfection using Lipofectamine RNAiMAX (Invitrogen, USA). To prepare the siRNA transfection solution for each tube, 20 pmol of siControl or siPPP2R4 was mixed with 50 μL of Opti-MEM reduced-serum medium by gentle pipetting. In parallel, 1.5 μL of Lipofectamine RNAiMAX was mixed with 50 μL of Opti-MEM. The two solutions were mixed by gentle pipetting and incubated for 10–20 min at room temperature to allow siRNA/lipid complexes to form. EC cells were suspended in complete growth medium without antibiotics to 50,000 cells/mL, then gently mixed with 100 μL of the transfection solutions, and plated. The cells were incubated for 24–72 h at 37°C and then assayed for gene knockdown.

The siRNA sequences used were 5’-GCAGUUCGCAGCUGAUAGA-3’ 5’-UGGAGUGUAUCCUGUUUAU-3’, 5’-GAUGAAGACUGGCCCAUUU-3’, 5’-CCAACCAGCUGUGGAACAU-3’ (siPPP2R4), and 5’-UGGUUUACAUGUCGACUAA-3’ (siControl).

### Cell proliferation assay

The cells transfected with the siRNAs were seeded at 5,000 cells per well in 96-well plates and incubated in a medium containing 10% FBS. After three days, the WST-8 reagent (Cell Counting Kit -8; Dojindo Molecular Technologies, Inc., Kumamoto, Japan) was added to each well, and the plates were incubated at 37°C for 1 h. Absorbance was measured at 570 nm using an automated microplate reader (Infinite 200; Tecan, Männedorf, Switzerland).

### Caspase assays

Caspase activity was measured 72 h after the siRNA transfection using the Caspase-GIO 3/7 assay (Promega, Madison, WI, USA) according to the manufacturer's instructions. Cells (5,000 cells/well) were seeded in 96-well white plates and incubated for 72 h. The caspase-3/7 substrate was then added to each well for 1 h at room temperature. Luciferase activity was measured using a microplate luminometer (Infinite 200; Tecan). The luminescence values were divided by the WST-8 absorbance values to correct for the differences in cell numbers and to calculate the luminescence per cell.

### Western blot analysis

Cells were lysed in complete lysis-M buffer (Roche Applied Science, Tokyo, Japan) containing the Halt phosphatase inhibitor cocktail (Thermo Fisher Scientific, Inc., Wayne, MI, USA). Lysates (10 μg of protein) were resolved using 10% sodium dodecyl sulfate polyacrylamide gel electrophoresis, and the proteins were transferred to nitrocellulose membranes (GE Healthcare Japan, Tokyo, Japan). The primary antibodies were diluted (1:2,000 for phospho-ERK1/2 and ERK1/2; 1:1,000 for phospho-GSK3βand GSK3β; 1:1,000 for phospho-rpS6 and rpS6; 1:1,000 for cyclin D1; 1:1,000 for phospho-AKT and AKT; and 1:5,000 for β-actin) and incubated with the membranes overnight at 4°C. The secondary antibodies [enhanced chemiluminescence (ECL) HRP-conjugated anti-rabbit IgG and anti-mouse IgG; GE Healthcare] were incubated with the membranes at room temperature for 60 min. Signals were detected using the ECL Select western blotting detection kit (GE Healthcare). Signal intensity was quantified using a densitometer (CS Analyzer version 3.0 software; ATTO, Tokyo, Japan) and normalized to the β-actin levels.

### Statistical analysis

Statistical analysis for the cell proliferation assay was performed using an independent *t*-test, Mann Whitney-U test, or Kruskal-Wallis H test. Comparisons between paired values were made using the Wilcoxon signed-rank test. All comparisons were performed using a two-sided test. Means, 95% confidence intervals (95% CIs), standard errors, and standard deviations were calculated for continuous variables. A *P* value < .05 was considered statistically significant. All statistical analyses were performed using the SPSS software (version 23; IBM SPSS, Inc., Chicago, IL, USA). The post-hoc power of the study was calculated using the G*Power software (version 3.1.9.2; free software). The statistical log file is provided as supplementary data.

## Results

### Patients’ characteristics

**Fig 1:**
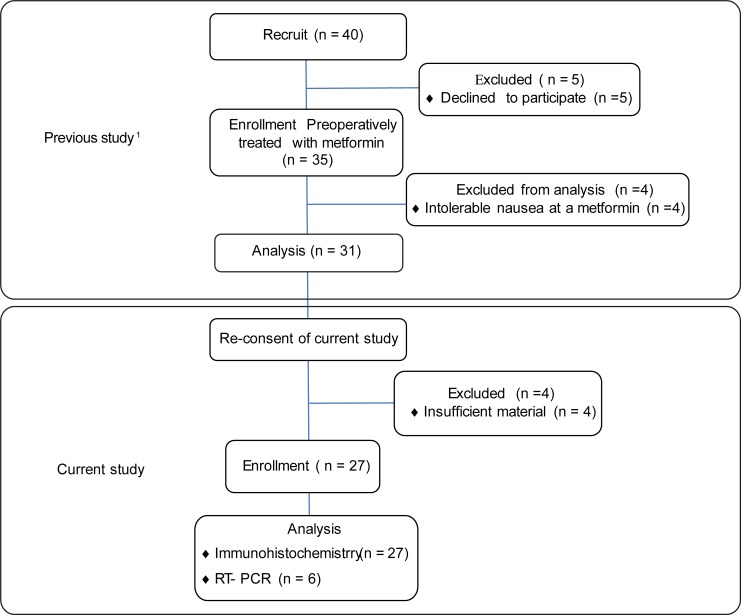
Flowchart of patients. ^1^ This study is registered with UMIN000004852. The results of this study are published in *Cancer 2014;120(19)*:*2986–95*.

The flowchart of the patients is shown in Fig 1. A total of 27 patients were included in the current study, and the patient characteristics are listed in Table 1. Eighteen patients (66.7%) had abnormal glucose metabolism. Seventeen patients (63.0%) were insulin-resistant as indicated by a homehostasis model assessment of insulin resistance score of ≥ 2.5 (mean score: 3.20; range: 0.6–8.18). Twenty five patients (92.6%) had abnormal glucose metabolism or insulin resistance or obesity.

**Table 1:** Patient characteristics.

Age(years), median (range)		49 (28–72
Histology, No. (%)		
	Endometrioid carcinoma grade 1	20 (74.1)
	Endometrioid carcinoma grade 2	6 (22.2)
	Mix carcinoma	1 (3.7)
Stage^a^, No. (%)		
	I	21 (77.8)
	II	1 (3.7)
	III	5 (18.5)
75 g OGTT, No. (%)		
	Normal	9 (33.3)
	IGT	11 (40.7)
	DM type	7 (25.9)
BMI, mean (range)		28.1 (16.5–50.5)
	≥ 25, No. (%)	18 (66.7)
HOMA-IR, mean (range)		3.20 (0.64–8.08)
	≥ 2.5, No. (%)	17 (63.0)

Abbreviations: BMI, body mass index; DM, diabetes mellitus; HOMA-IR, homeostasis model assessment of insulin resistance; IGT, impaired glucose tolerance; OGTT, oral glucose tolerance test.

^a^ The International Federation of Gynecology and Obstetrics (FIGO) stage.

### Metformin reduced PP2A expression in endometrial cancer tissues

**Fig 2:**
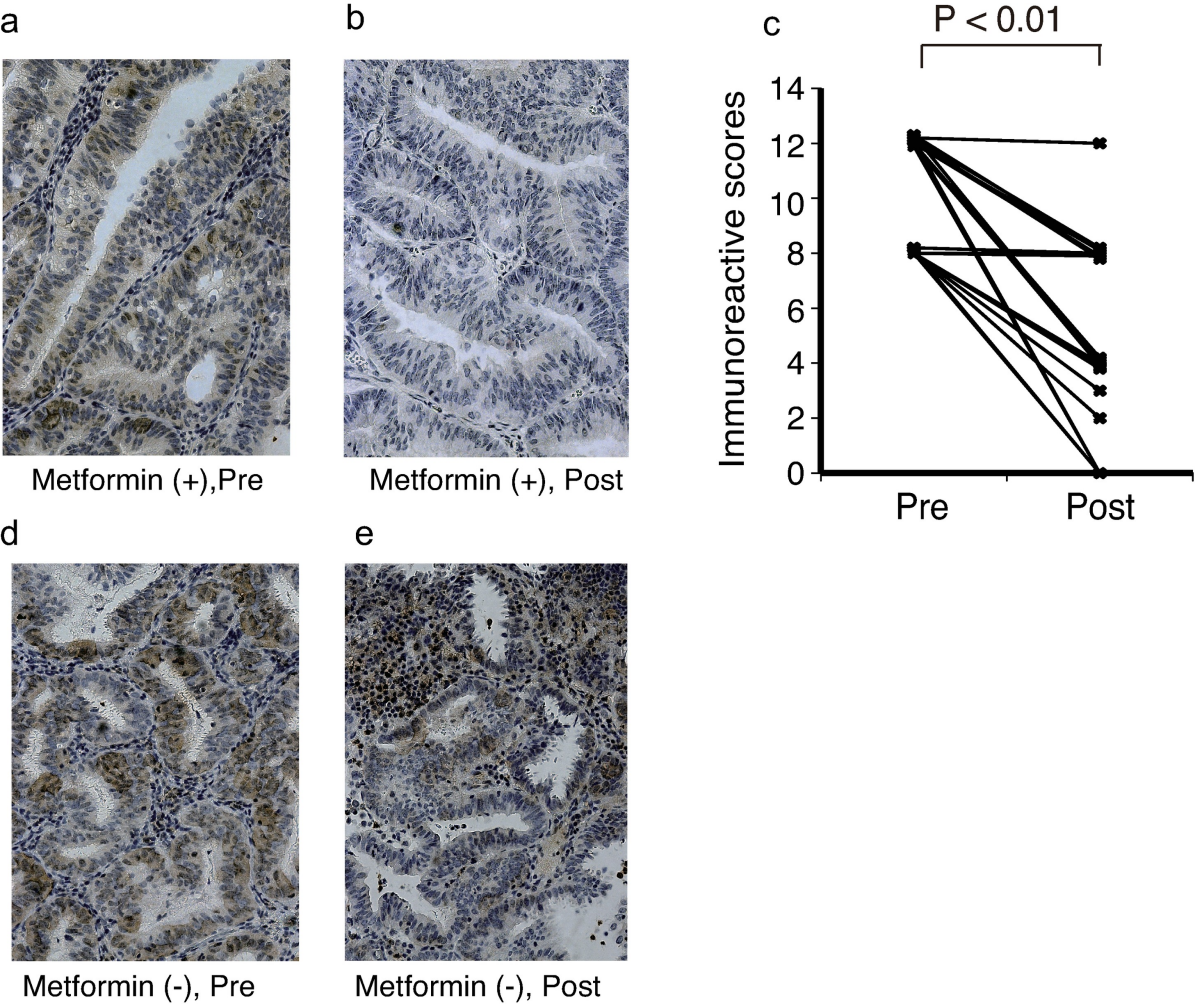
Preoperative metformin administration decreased immunostaining of PP2A in endometrial cancer tissues. Representative images of changes in immunostaining are shown for paired specimens obtained before (a) and after (b) metformin treatment. Differences in immunoreactive scores were evaluated using the Wilcoxon signed-rank test (c). Representative images of changes in immunostaining are shown for paired specimens obtained from preoperative biopsy (d) and hysterectomy (e) from patients who did not receive metformin treatment during the study period. Pre, sampling at the time of diagnosis in each group and before the initiation of metformin treatment in the metformin treatment group; Post, sampling immediately before the operation in each group and after metformin treatment in the metformin treatment group.

We investigated the effect of metformin on the expression of PP2A in EC tissues. The 27 patients who were treated with metformin preoperatively were evaluated for PP2A-B expression using IHC. The expression of PP2A in the EC tissues was higher than that in the normal endometrium specimens. The mean IRSs of the PP2A-B expression level of normal endometrium was 1.5 [95% confidence interval (CI), 0.24–2.76]. Preoperative metformin treatment resulted in significantly reduced PP2A expression in 22 patients (81.5%), while the expression did not change in five patients (Fig 2A and 2B). The mean IRSs of the PP2A-B expression level was reduced from 10.2 (95% CI, 9.4–11.0) to 5.1 (95% CI, 3.8–6.3; *P* < .01) (power 1.0) (Fig 2C). To confirm that this finding did not result from a natural change that occurred between the two sampling times, we compared 10 additional EC patients who did not receive metformin. The IRSs of the PP2A-B level varied from 8.2 (95% CI, 5.8–10.6) to 7.2 (95% CI, 5.5–8.9), i.e., no significant changes in the PP2A expression were observed in these cases (Fig 2D and 2E).

**Fig 3:**
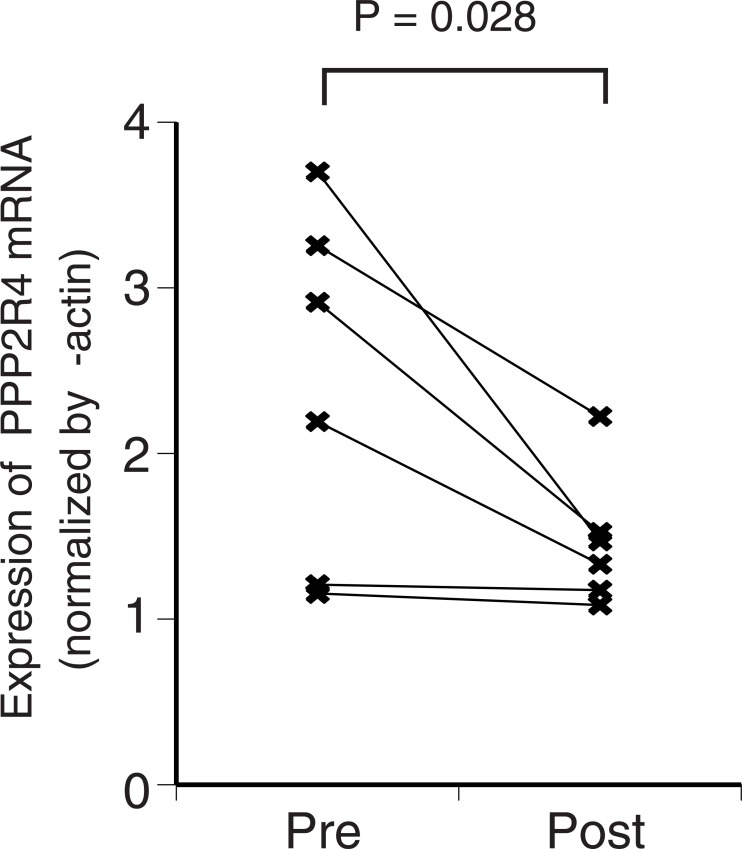
Preoperative metformin administration significantly reduced the *PPP2R4* mRNA expression in endometrial cancer tissues. The *PPP2R4* expression in endometrial cancer tissues was evaluated using real-time polymerase chain reaction, and the values were normalized to those of β-actin. The intensity of *PPP2R4* mRNA expression was based on that of the normal endometrium (n = 6) (Wilcoxon-signed rank test). Pre, before the initiation of metformin treatment; Post, after metformin treatment.

Additionally, we evaluated the effect of metformin on *PPP2R4* mRNA expression in EC tissues. Using paired samples from six patients (before and after metformin treatment), we examined the changes in the *PPP2R4* mRNA expression in EC tissue, caused by the administration of metformin. The *PPP2R4* expression in EC tissues was higher than that in the normal endometrium. Metformin administration resulted in significantly reduced *PPP2R4* mRNA expression (mean proportional decrease of 31.3%; 95% CI, 7.2–55.3; *P* = .028) (power 0.77) (Fig 3).

These results indicate that metformin administration downregulated PPP2R4 expression in patients with endometrial cancer.

### Metformin does not directly affect PP2A in vitro

**Fig 4:**
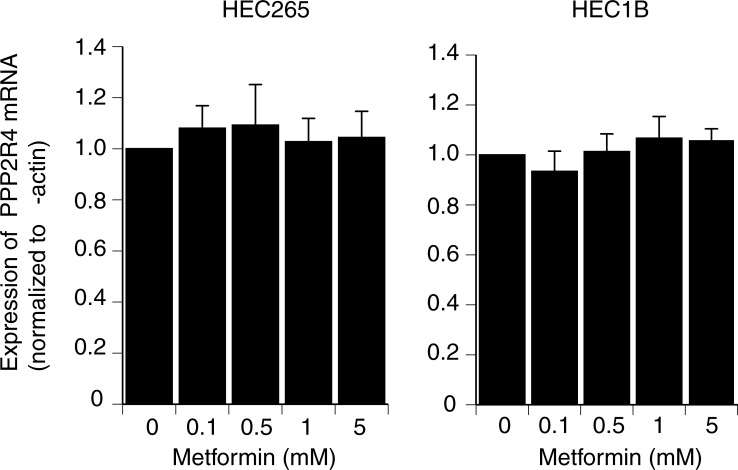
Effects of metformin on *PPP2R4* mRNA levels *in vitro*. HEC 265 and HEC 1B were seeded in 6-well plates (50,000 cells/well) in Dulbecco’s modified Eagle’s medium containing 10% fetal bovine serum. At 80% confluency, the cells were treated with increasing doses (0.1–5 mM) of metformin for 24 h. There were no significant changes in the expression of *PPP2R4* compared with the control at any concentration of metformin in either endometrial cancer cell line. The results are presented as the mean ± standard deviation for three independent experiments.

To examine whether the metformin effect was direct or indirect, we performed an *in vitro* assay using the EC cell lines (HEC265 and HEC1B) stimulated with metformin. There were no significant changes in the expression of *PPP2R4* at any concentration of metformin in both cell lines (Fig 4). Metformin could not directly reduce the *PPP2R4* mRNA expression. Therefore, it was considered that metformin indirectly inhibited the *PPP2R4* expression.

### Anticancer effects of PP2A inhibition in endometrial cancer cell lines

**Fig 5:**
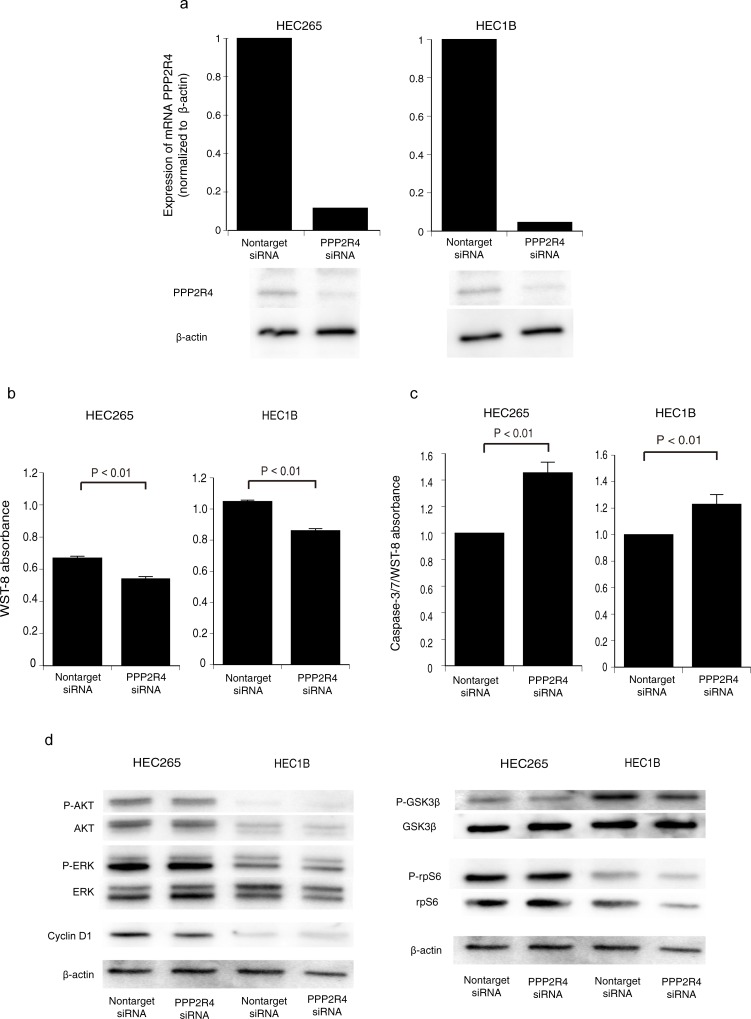
Effects of siRNA knockdown of *PPP2R4*. (a) Real-time polymerase chain reaction and western blot analysis of cancer cells transfected with *PPP2R4*-specific siRNA and control siRNA. (b) WST-8 assay using cancer cell lines transfected with the *PPP2R4* siRNA and control siRNA. (c) Caspase 3/7 activity in the cancer cell lines transfected with the *PPP2R4* siRNA and control siRNA. (d) Western blot analysis of cell signaling changes in endometrial cancer cells transfected with the PPP2R4 siRNA and control siRNA. The expression levels of phospho-extracellular signal-regulated kinase 1/2 (ERK1/2), ERK1/2, phospho-protein kinase B (AKT), AKT, phosphor-ribosomal protein S6 (rpS6), rpS6, phospho-glycogen synthase kinase-3 beta (GSK3β), GSK3β, and cyclin D1 in cancer cells transfected with the siRNAs are shown. Columns and error bars represent the mean ± the standard error of the mean for at least three independent experiments.

To explore the role of PPP2R4 in EC cell lines, we knocked down *PPP2R4* using a siRNA. HEC265 and HEC1B cells were selected because they are model cell lines of grade 1–2 endometrial carcinoma. The PPP2R4 knockdown efficiency of the gene-specific siRNA was confirmed using real-time PCR and western blot. *PPP2R4* expression in endometrial carcinoma cell lines (HEC265 and HEC-1B) was inhibited at both protein and mRNA levels by transfection with the *PPP2R4*-specific siRNA (Fig 5A). The knockdown of *PPP2R4* gene expression with the siRNA significantly reduced the proliferation rates of HEC265 (*P* < .01) and HEC1B (*P* < .01) cells compared with those of the cells transfected with the non-targeting siRNA (Fig 5B). Additionally, the knockdown of *PPP2R4* increased the caspase activity of HEC265 (*P* < .01) and HEC1B (*P* < .01) cells (Fig 5C), which was consistent with the results of the WST-8 assay. These results suggested that downregulation of *PPP2R4* in HEC265 and HEC1B cells inhibited the cell proliferation and induced apoptosis.

PP2A inhibitors such as okadaic acid increase the AKT activity [32], which is one of the reasons why PP2A is considered a tumor suppressor. Therefore, we examined the possibility that a PPP2R4 knockdown is associated with the cancer proliferation via the MAPK and AKT /m TOR pathway regulation in EC cells. There were no significant differences between the PPP2R4 knockdown and control cells in the expression of ERK, AKT, rpS6 and cyclin D1(Fig 5D).

We found significantly decreased levels of phospho-GSK3β, which were quantified by densitometry and normalized to β-actin. siRNA knockdown of *PPP2R4* in HEC265 (47%) and HEC1B (66%) cells decreased the phospho-GSK3β/GSK3β ratio significantly compared to cells transfected with non-targeting siRNA (Fig 5D).

## Discussion

In this study, we found that an antidiabetic dose of metformin could reduce the expression of PP2A in patients with EC and demonstrated that *PPP2R4* knockdown led to the inhibition of cell proliferation and to the induction of apoptosis in EC cells. Thus, PP2A inhibition was considered one of the antiproliferative effects of metformin administration.

The reduction of PP2A expression was related to the antiproliferative effect of metformin in the EC patients. However, this *in vivo* antiproliferative effect was considered to be indirect based on the fact that the *in vivo* metformin concentration was too low to inhibit the cell growth *in vitro* [10]. Hormonal factors such as insulin-like growth factor 1 (IGF-1) and leptin have been shown to be decreased by metformin administration [10] and may potentially be responsible for the reduction of the cancer growth-supporting potential of patient sera. Preclinical data in animal models have also suggested that the antitumorigenic effects of metformin may depend on the metabolic composition of the host. Metformin has been found to be more effective in inhibiting tumor growth in obese and insulin-resistant animals than in their lean counterparts in breast and lung cancer models [33, 34]. In a mouse breast cancer model, metformin could suppress the obesity-induced secretion of adipokines as well as breast tumor formation and growth. Metformin also suppressed the secretion of IGF-1, IGF-2, leptin, and the tissue metallopeptidase inhibitor 1 and decreased the lipid accumulation during adipocyte differentiation [35]. Furthermore, our findings showed that metformin did not reduce PPP2R4 expression in EC cell lines *in vitro*. These results led us to conclude that metformin indirectly reduced the expression of PP2A and subsequently induced the antiproliferative effect in EC patients.

Some reports have suggested that inhibition of PP2A has the potential as a cancer treatment [17, 23, 24, 36–38]. The small molecule inhibitor LB100 exhibited potential antineoplastic activity, in combination with cisplatin, in an intracranial xenograft model [25, 38]. This suggests that some inhibitors of PP2A may inhibit the cancer cell growth. siRNA knockdown of *PPP2R4* led to the apoptosis and growth inhibition of HeLa cells [26, 27] and induced the apoptosis in the HEK 293 and N2a cell lines through a mitochondrial pathway [39]. However, one study reported that both inhibition and overexpression of PPP2R4 induced cell death in human and opossum cells [28]. PPP2R4 is necessary for cell homeostasis. In addition to PPP2R4, inhibition of other PP2A subunits is considered a potential cancer treatment. PP2A inhibition by okadaic acid significantly enhanced the response to lapatinib in breast cancer cells via decreased phosphorylation of eukaryotic translation elongation factor 2 [40]. Downregulation of the *PPP2R5C* gene expression might be considered as a new therapeutic target strategy for chronic myeloid leukemia [41]. PP2Ac upregulation showed a poor prognostic impact on the overall survival of hepatocellular carcinoma (HCC) patients, and PP2Ac downregulation was shown to be a potential therapeutic target for HCC [18, 19]. Thus, PP2A inhibition might be considered for the treatment of some malignancies.

There is currently a controversy regarding whether PP2A functions as a tumor activator or tumor inhibitor. PP2A has been considered a tumor suppressor based on the fact that activation of PP2A leads to tumor growth inhibition [16]. Okadaic acid, a potent inhibitor of PP2A, and several endogenous inhibitors of PP2A have been shown to promote the malignant growth of human cancer by increasing the expression of ERK and AKT [17]. Inhibition of PP2A activity using a small hairpin RNA lead to the suppression of several B-subunits and induced cell transformation, suggesting that PP2A might act as a tumor suppressor. The functions of PP2A in cancer are diverse, and its effects are different depending on the regulatory subunits involved. The B/56β and B/56γ subunits are known to dephosphorylate ERK [42], while B/55α-dependent PP2A acts as a regulator of AKT signaling [43]. Based on our results, siRNA knockdown of *PPP2R4* did not affect the ERK and AKT pathway in EC. However, siRNA knockdown of *PPP2R4* increased GSK3β activity by dephosphorylation at Ser 9. Oncogenic *PPP2R1A* mutation, which promotes cancer proliferation, decreased GSK-3 activity by phosphorylation at Ser 9 [20]. GSK-3 can suppress the Wnt/β-catenin pathway by phosphorylating β-catenin, resulting in ubiquitin/proteasome-dependent degradation of β-catenin [44]. GSK-3 serves as a potential tumor suppressor candidate through *PPP2R4* inhibition. Thus, we speculated that PPP2R4 inhibition leads to an antitumor effect rather than to cancer cell proliferation in EC. Extensive molecular analysis is needed to elucidate the mechanism of the antitumor effect of PPP2R4 inhibition in EC.

This study had some limitations. First, we used metformin mainly for grade 1 and 2 EC. Type 1 EC tends to be associated with obesity, insulin resistance, and type 2 diabetes mellitus. Therefore, the frequency of obesity or insulin resistance was inevitably increased in this study and led to a bias. It is not clear whether metformin has antitumor effects in patients with type 2 EC (serous, clear endometrioid carcinoma of grade 3) and in lean patients. Second, PP2A has 92 different holoenzymes [44], however, we did not investigate the other PP2A subunits. To further characterize the function of metformin in relation to PP2A, more data on each holoenzyme are needed.

In conclusion, we found that metformin indirectly downregulated PPP2R4 in EC patients, and PPP2R4 inhibition led to an antitumor effect on EC cells *in vitro*. Our findings show the potential mechanisms underlying the anticancer effect of metformin. PP2A inhibition might be considered a target for the treatment of EC patients with insulin resistance.

## Supporting information

S1 File TREND statement. (PDF)

S2 File Protocol original language. (PDF)

S3 File Protocol UMIN 000004852. (PDF)

S1 Fig The statistical log file of analysis about IHC staining of PP2A-B in the EC tissues. Differences in immunoreactive scores between paired specimens obtained at the time of preoperative biopsy and at surgery were evaluated using an independent *t*-test and the Wilcoxon signed-rank test (Fig 2C).
(PDF)

S2 Fig The statistical log file of analysis about the *PPP2R4* expression in EC tissues. Differences between paired specimens obtained at the time of preoperative biopsy and at surgery were evaluated using the Wilcoxon signed-rank test (Fig 3).
(PDF)

S3 Fig The statistical log file of analysis about WST-8 assay in HEC 265 cells. Differences between cancer cell lines transfected with the *PPP2R4* siRNA and control siRNA were evaluated using an independent *t*-test and the Mann-Whitney U Test (Fig 5B).
(PDF)

S4 Fig The statistical log file of analysis about WST-8 assay in HEC IB cells. Differences between cancer cell lines transfected with the *PPP2R4* siRNA and control siRNA were evaluated using an independent *t*-test and the Mann-Whitney U Test (Fig 5B).
(PDF)

S5 Fig The statistical log file of analysis about caspase 3/7 activity in HEC 265 cells. **(**Differences between cancer cell lines transfected with the *PPP2R4* siRNA and control siRNA were evaluated using an independent *t*-test and the Kruskal-Wallis test (Fig 5C).
(PDF)

S6 Fig The statistical log file of analysis about caspase 3/7 activity in HEC 1B cells. Differences between cancer cell lines transfected with the *PPP2R4* siRNA and control siRNA were evaluated using an independent *t*-test and the Kruskal-Wallis test (Fig 5C).
(PDF)
