# Supplementary material for: Antitumor effects of metformin via indirect inhibition of protein phosphatase 2A in patients with endometrial cancer
Source: PLoS One. 2018 Feb 14;13(2):e0192759. doi: 10.1371/journal.pone.0192759 (PMC5812621; doi:10.1371/journal.pone.0192759)
Supplement: S2 File — (PDF) [file pone.0192759.s002.pdf]

自主臨床試験課題名：

「子宮体癌に対するメトホルミンの効果に対する探索的試験:
細胞周期蛋白・ERK/AMPK-mTOR 経路関連蛋白に対する効果の検討」

試験責任者：千葉大学医学部付属病院婦人科　　　准教授　三橋　暁

実施場所：　千葉大学医学部附属病院婦人科
〒260-8677 千葉市中央区亥鼻 1-8-1
TEL：043-222-7171 内線（6611）
FAX：043-26-2342

緊急連絡先：TEL：043-222-7171 内線（6611）

臨床試験実施期間：平成 22 年承認後 ～ 平成 26 年 12 月 31 日

平成 24 年 7 月 24 日 計画書 第二版作成
平成 25 年2月13日 計画書 第三版作成
平成 26 年10月 20 日 計画書 第四版作成

目次

1.試験の背景

2.試験の目的と必要性

3.試験薬の概要

4.対象患者

4-1 選択基準
4-2 除外基準

5.被験者に説明し同意を得る方法

6.試験の方法

6-1 試験の種類・デザイン
6-2 試験のアウトライン
6-3 被験者の試験参加予定期間
6-4 研究方法
6-5 副作用による投与の変更基準
6-6 投与期間
6-7 被験者ごとの中止基準
6-8 併用禁止薬
6-9 症例登録
6-10 試料等の保存方法およびその必要性
6-11 試料等の廃棄方法およびその際の匿名化の方法

7.評価項目

7-1 主要評価項目
7-2 副次的評価項目

8. 安全性の評価

8-1.有害事象判定基準
8-2. 予想される副作用

9.観察、検査、調査項目およびその時期

9-1. 検査、調査項目およびスケジュール
9-2. 患者背景
9-3 臨床検査(血液学的検査、血液生化学検査)
9-4 臨床所見(自覚症状・他覚症状)
9-5 安全性に関する観察期間
9-6 有効性に関する観察期間

10.有害事象の取り扱いについて

10-1 有害事象発生時の被験者への対応
10-2 重篤な副作用の報告

11.試験全体の中止または中断

12.目標症例および研究期間

12-1 目標症例数
12-2 設定根拠
12-3 研究期間

13.データマネジメントおよび解析方法

13-1 データマネジメント
13-2 解析対象集団
13-3 評価項目、方法

14. 被験者の人権および安全性・不利益に対する配慮ならびに個人情報保護

14-1 人権への配慮(プライバシーの保護)
14-2 安全性、不利益への配慮
14-3 個人情報保護

15. 患者の費用負担

16. 健康被害の補償および保険への加入

17. 臨床研究に関する倫理指針 およびヘルシンキ宣言への対応

18 研究資金及び利益の衝突

19.研究組織
20.研究施設
21.参考文献

1. 試験の背景

脂肪の過剰摂取など食事の欧米化・晩婚化・出産回数の低下などライフスタイルの変化に伴い、本邦における子宮体癌発生頻度は年々増加している。日産婦婦人科腫瘍委員会の子宮体癌 I–IV 期の登録数は、1994 年の 2115 例から 2005 年の 4267 例に増加している [1)]。 特に肥満との関係が近年指摘され、子宮体癌の 40%は肥満と関連しているとの報告がある [2)]。

一方、メトホルミンは糖尿病の治療薬として広く使用されている薬剤であるが、近年メトホルミン投与中の糖尿病患者に癌の発癌が少ないことが報告され、疫学的に癌の予防効果が指摘された [3)]。その後乳癌、大腸癌、前立腺癌、卵巣癌培養細胞株を用いた観察で、メトホルミン投与により増殖抑制効果が報告され [4–5)]、抗腫瘍効果も期待される薬剤である。またメドロキシプロゲステロン（MPA）抵抗性の子宮内膜増殖症への効果も報告もされた [6)]。乳癌ではメトホルミン服用例で術前化学療法の奏功率が良好との報告があり、メトホルミン投与の臨床試験もはじまっている。

我々は子宮内膜癌培養細胞株に対するメトホルミンの効果を検証し、子宮内膜癌培養細胞株の増殖抑制効果を確認した。この増殖抑制作用機序として、AMPK の活性化・ERK の抑制による G1 停止と考えられる（未発表）。ホルモン依存性子宮体癌症例は糖尿病合併例や耐糖能異常症例が多いことがしられ、我々は、インスリン抵抗性による高インスリン血症が、子宮体癌の発癌過程に関与していると推測している。

そこで本試験では、メトホルミンの子宮体癌細胞への直接効果を確認することを目的とする。メトホルミンは糖尿病の治療薬として安全性が確立していること、海外では耐糖能異常症例での糖尿病予防効果も報告されている薬剤である [7)]。 子宮体癌患者で耐糖能異常を有する症例に、手術待機までの期間メトホルミンを投与し、細胞周期蛋白、増殖シグナルの変化を検証する。今回の探索的試験で、子宮体癌細胞に対する増殖抑制効果が確認されれば、今後再発予防や、再発例に対する治療など、臨床応用も期待される。ホルモン依存性子宮体癌症例は糖尿病合併例や耐糖能異常症例が多いことがしられ、メトホルミン服用による副次的な糖尿病の予防効果も期待できる。

2.試験の目的と必要性

培養細胞を用いた研究で、メトホルミンの子宮体癌に対する増殖抑制効果が確認された。本試験では、子宮体癌細胞に対するメトホルミンの直接効果を確認する。短期的な投与で、細胞周期蛋白・ERK/AMPK-mTOR 経路関連蛋白への作用を確認する。子宮体癌細胞への効果が確認されれば、今後再発予防効果や、抗がん剤との併用、さらに再発症例に対する臨床効果も期待できる。

3.試験薬の概要

3-1 メトホルミン

4.対象患者

次の選択基準を満たし、除外基準に当てはまらない患者を対象とする。

4-1.選択基準

(1) 子宮体部類内膜腺癌(高分化型・中分化型)と診断され手術予定の患者。術前検査で耐糖能異常が指摘された症例
(2) 年齢 20 歳以上、70 歳未満
(3) 一般全身状態 PS 0
(4) 血清ｸﾚｱﾁﾆﾝ 0.8mg/dl 以下
(5) 本治療使用薬剤に対し過敏症の既往歴のない症例
(6) 患者本人から文書にて同意の得られた症例

4-2.除外基準

本試験の登録にあたっては、以下のいずれの基準にも抵触してはならない。

(1) 本試験使用薬剤に対して重篤な過敏症、あるいは薬物アレルギーの既往を有する症例
(2) 乳酸アシドーシスの既往
(3) 透析患者
(4) ショック、心不全、心筋梗塞、肺塞栓など心血管系の傷害
(5) 過度のアルコール摂取
(6) 肝機能障害
(7) 血栓の既往
(8) 抗精神薬で治療中または治療を要すると思われる精神障害を有する症例
(9) すでに糖尿病で治療している症例・新たに糖尿病が見つかり薬物治療が必要な症例
(10) 活動性の重複癌を有する症例
(11) その他、主治医が不適格と判断した症例。

5.被験者に説明し同意を得る方法

試験責任(担当)医師は、千葉大学医学部附属病院治験審査委員会での承認の得られた同意説明文書を患者さんに渡し、文書および口頭により十分な説明を行い、患者さんの自由意思による同意を文書(記名・捺印または署名し、同意年月日を記入する)にて得る。同意文書には説明をおこなった医師の記名・捺印し、その日付を記入する。

患者さんの同意に影響を及ぼすと考えられる有効性、安全性等の情報が得られたときや、患者さんの同意に影響を及ぼすような実施計画等の変更が行われるときは、速やかに患者さんに情報提供し、試験等に参加するか否かについて患者さんの意思を予め確認するとともに、事前に治験審査委員会の承認を得て同意説明文書等の改訂を行い、患者さんの再同意を得ること。

本試験の実施にあたり、担当医は下記の事項に関して「説明文書と同意書」を用いて患者及び家族等に手渡して口頭で説明する。

患者に対する説明事項

1. はじめに: 自主臨床試験について、あなたの病気について
2. この試験の目的
3. この試験の方法
4. この試験の予定参加期間
5. この試験薬の予想される効果と起こるかもしれない副作用
6. この薬を使用しない場合の、他の治療方法
7. この試験中に、あなたの健康に被害が生じた場合について
8. この試験への参加は、患者さんの自由意思によるものです
9. この薬に関する情報は、随時ご連絡します
10. この薬の使用を中止させていただく場合があります
11. この試験に参加された場合、あなたのカルテなどが試験中あるいは試験終了後に調査されることがあります
12. この試験結果が公表される場合でも、あなたの身元が明らかになることはありません
13. この試験への参加に同意された場合に守っていただくこと
14. あなたの費用負担について
15. 担当医師について
16. 相談窓口について

説明後、本試験への参加について、患者本人の自由意志による同意を説明文付き同意書で得る。その際には、患者に 1 日以上の考慮する時間を与え、患者本人が良く理解したことを確認した上で、参加の依頼を行う。同意文書は 1 部を患者へ渡し、もう一部をカルテに保存する。

6.試験の方法

6-1 試験の種類・デザイン

探索的臨床試験

6-2 試験のアウトライン

症例選択基準に合致する患者に同意を得たのち、研究事務局に登録適格性確認票を提出する。適格性が確認されたのち登録番号が発行され、投与開始する。試験薬の投与は、手術後 1 か月目まで行う

6-3 被験者の試験参加予定期間

試験期間は、症例選択基準に合致することを確認し、同意取得から手術までの期間

6-4 試験方法

I. 試験方法

1) 患者背景(身長、体重、BMI)を確認。耐糖能異常が指摘された症例で 75g OGTT 検査施行し、前値、負荷後 30 分、60 分、120 分の血糖、IRI を測定する。また HOMA-R を計算する。

2)メトホルミン 750mg　分3　毎食後　内服投与開始、悪心・嘔吐・血液毒性・臨床検査値異常が出現しなければ一週毎に 750mg/日増量し 2250mg/日まで増量する。増量後に副作用出現した場合は減量する。 メトホルミンは登録後、手術終了後 1 か月間継続する(周術期は手術前日まで服用、食事開始後再開)

3)検体採取

子宮体部類内膜腺癌の診断的内膜検査時に採取した組織を分割し、病理検査に提出。類内膜腺癌と診断された組織を治療前の組織として解析。メトホルミン服用後は手術時に組織採取し治療後組織として解析

治療開始時、検査時、治療終了時の血清を採取、-80 度で凍結保存

4) メトホルミン服用前後の組織より蛋白を抽出、ウエスタンブロット法にて S6K1, リン酸化 S6K1, ERK1/2, リン酸化 ERK1/2,　AKT, リン酸化 AKT,　pRb, リン酸化 pRb ,cyclin D1, p27, AMPK, リン酸化 AMPK の蛋白発現量を検討する

5)免疫組織学的検討により上記蛋白のほか、BCL-2, ki-67, TOPO II の発現も検討する

6) 治療前後の血清を用いた培養実験。子宮内膜癌培養細胞株 ISHIKAWA 株を 2%患者血清入りの DMEM で培養し、細胞増殖を確認する。

II. 評価項目、方法

1) メトホルミン投与前後で蛋白発現の差を検討。CS Analyzer による解析で比較す

2) 免疫組織学的に陽性細胞の頻度を比較

3) 空腹時血糖、空腹時インスリン、 IGF-1, レプチン、アディポネクチン、HOMA-R, 体重の変化を確認　耐糖能異常の変化を検討

4)細胞増殖は、thymidine の取り込みで評価する。治療前後の血清で増殖が異なるか検討

6-5 副作用による投与の変更基準

・以下の臨床検査異常がでた場合は投与中止。

総ﾋﾞﾘﾙﾋﾞﾝ	1.5mg/dl 以上
BUN	25mg/dl 以上
血清ｸﾚｱﾁﾆﾝ	1.0 mg/dl 以上
grade 2 以上の血液毒性	

- 悪心・嘔吐が出現した場合は一旦服用中止。臨床検査異常がない場合は一段階減量して再開し、悪心・嘔吐が出現しない場合は継続する。
- grade 2 以上の下痢

6-6 投与期間

原則として「6-7 被験者ごとの中止基準」に抵触しない限り投与を継続する。
登録後、手術終了後 1 か月間メトホルミン服用する

6-7 被験者ごとの中止基準

以下の場合は、試験責任（担当）医師の判断で試験を中止する。

(1) 有害事象が発現した場合
(2) 合併症の悪化などにより病態が急激に不良となった場合
(3) 患者または家族から試験中止の申し出があった場合
(4) その他、試験責任（担当）医師が本試験の継続を不可能と判断した場合

6-8 併用禁止薬

メトホルミン投与から手術までの期間、抗がん剤、ホルモン剤の服用は禁止する。メトホルミン以外の糖尿病治療薬も禁止する（治療を要する症例は除外）

6-9 症例登録

患者の登録はインフォームドコンセントを得ておこなう。試験責任（担当）医師は対象患者が適格基準を満たし、除外基準のいずれにも該当しないことを確認し、文書による同意を得た後、登録適格性確認用紙に必要事項をすべて記入のうえ、研究事務局に登録適格性確認票を提出する。適格性が確認されたのち、登録番号が発行される。研究事務局より試験責任（担当）医師に連絡する。

6-10 試料等の保存方法およびその必要性

本臨床研究に係わる診療記録、検査データ、患者の同意に関する記録など医療機関において作成されたものについては各医療機関において保管責任者が適切に保管する。保管期間は本研究終了時までとする。なお、試料は、患者の同意が得られた場合には、各研究機関において、事務局の責任において10年間は保存する。また新たに解析項目を加える場合、必ず新たに倫理委員会の承認を得た後に行う。

6-11 試料等の廃棄方法およびその際の匿名化の方法

匿名化されている試料等については、次のように廃棄する。蛋白についてはチューブのラベルをはがすか内容物を除去した後、廃棄する。書類などは焼却処分し、コンピューターの外部記憶装置に保存していた情報は消去する。個人識別情報については、個人情報管理者がコンピューターの外部記憶装置に保存していた情報を消去する。

7.評価項目

7-1 主要評価項目

メトホルミンの子宮体癌への直接効果を検討する
①メトホルミン投与前後での MAPK 経路、m TOR 経路、AMPK 経路、細胞周期蛋白の変化 （S6K1,リン酸化 S6K1, ERK1/2, リン酸化 ERK1/2, AKT, リン酸化 AKT, pRb, リン酸化 pRb ,cyclin D1, p27, AMPK, リン酸化 AMPK）

②免疫組織学的検討により上記蛋白のほか、BCL-2, ki-67、TOPO II の発現の変化

7-2 副次評価項目

メトホルミン投与後の HOMA-R の変化, インシュリン、IGF-1, レプチン、アディポネクチンの変化を確認

投与前後の血清を用いた増殖抑制効果の確認

8. 安全性の評価

8-1.有害事象判定基準

臨床検査値、臨床所見(自他覚所見)に関する有害事象を以下に従い評価する。

(1)有害事象の定義

有害事象とは、「医薬品(治験薬)が投与された際に起こる、あらゆる好ましくない、或いは意図しない兆候(臨床検査値の異常も含む)、症状、又は病気のことであり、当該医薬品(治験薬)との因果関係の有無は問わない」と定義される。投与前より異常が認められた場合は、当該事象の Grade が 1 段階以上悪化した時に有害事象として扱うこととする。

(2)重症度の分類

投与開始後に有害事象を観察した場合には、試験責任者(担当)医師は速やかに適切な処置を講ずるとともに、「NCI-CTC version2.0 JCOG 版」に従い重症度を分類し、症例報告書の該当する箇所に記載する。重症度分類以外の項目については、0:無、1:軽度、2:中等度、3:重度、4:重篤、の基準にて判定する。

8-2. 予想される副作用

悪心・嘔吐等消化器毒性

肝機能異常等臨床検査値異常

乳酸アシドーシス

低血糖

9.観察、検査、調査項目およびその時期

9-1. 検査、調査項目およびスケジュール

表 11-1-1 検査、調査項目およびスケジュール

観察時期	登録前※1	メトホルミン投与開始後				投与終了・中止後※2
		1w 目	2w 目	以後 2W 毎	手術前日	
患者背景	●				●	●
全身状態	●	●	●	●	●	●
臨床検査	●	●	●	●	●	●

●:必須検査

※1：登録前１４日～登録日とする。ただし、患者背景は登録前の日数は規定しない。画像検査および腫瘍マーカーに関しては登録前１４日前～登録日とする。
※2 ：中止・終了後、因果関係の否定できない有害事象が発現している場合は、原則として回復するまで観察を継続する。

9-2. 患者背景
以下の内容を登録前に調査・確認する。
登録時年齢、認識番号、身長、体重、癌腫、組織型、転移巣、PS、合併症の有無、既往歴、前治療内容および投与期間、同意取得日
耐糖能異常が指摘された症例で 75g OGTT 検査施行し、前値、負荷後 30 分、60 分、120 分の血糖、IRI を測定する。また HOMA-R を計算する。

9-3 臨床検査（血液学的検査、血液生化学検査）
① 血液学的検査
白血球、白血球分画（好中球）、血小板、赤血球、ヘモグロビン
② 生化学的検査
AST、ALT、γ-GTP, ALP、LDH、ビリルビン、UA、BUN、Cr、ALP、LDH、Na、K、Cl、glucose（空腹時）、insulin（空腹時）、HOMA-R, HbA1c（登録前異常者のみ）

9-4 臨床所見（自覚症状・他覚症状）
登録前、各受診時・中止時に、悪心、嘔吐、食欲不振、下痢、その他の異常所見の有無について観察をおこなう。

9-5 安全性に関する観察期間
安全性に関する観察・検査は原則として投与終了・中止後４週間までとする。ただし、試験薬との因果関係を否定できない有害事象が発現している場合は、原則として回復するまで観察を継続する。

9-6 有効性に関する観察期間
試験終了後も再発の有無に関して観察する。
後治療開始や転院により以後の観察が不可能となった場合はこの限りではない。

10.有害事象の取り扱いについて

10-1 有害事象発生時の被験者への対応
　本治療中に重篤あるいは未知の有害事象が発現した場合には、試験との関連性にかかわらず、担当医師はただちに適切な処置を講じ患者の安全確保に努めるとともに、カルテならびに症例報告書に齟齬なく記載する。また、試験薬の投与を中止した場合や、有害事象に対する治療が必要となった場合には、被験者にその旨を伝える。

10-2 重篤な副作用の報告

尚、重篤な副作用とは死亡、障害、もしくはそれにつながる恐れのある症状または事故であって、担当医の認めたものとする。

報告の対象となる有害事象：試験期間中の全ての重篤な有害事象、および試験終了（中止）後に試験薬との関連性が疑われる重篤な有害事象について報告すること。

試験責任医師は、重篤な有害事象の発生を認めたときは、速やかに病院長（臨床試験部）に報告する。報告は第一報（緊急報告）および第二報（詳細報告）とすること。厚生労働省の「医薬品・医療用具等安全性情報報告制度」により厚生労働省に報告する。

11.試験全体の中止または中断

下記の場合は本試験全体を中止する。試験中止が決定した場合、試験代表医師は、その旨を病院長に文書で報告する。

(1) 試験薬の品質、安全性、有効性に関する重大な情報が得られたとき。
(2) 被験者のリクルートが困難で、予定症例に達することが到底困難であると判断されたとき。
(3) 予定症例数または予定期間に達する前に、試験の目的が達成されたとき。
(4) 倫理委員会または治験審査委員会により、実施計画などの変更の指示があり、これを受け入れることが困難と判断されたとき。

12.目標症例および試験期間

12-1 目標症例数

目標症例数：40 症例

12-2 設定根拠

40 例/年の手術例が予想。

12-3 試験期間

試験期間は、症例集積期間 3 年とする。

症例集積期間:平成 22 年 4 月 26 日～平成 26 年 12 月 31 日

13.データマネジメントと解析方法

13-1 データマネジメント

13-1-1 データ収集

試験責任（分担）医師は、被験者のスクリーニング及び登録の記録を作成し、スクリーニングを実施した全被験者を記載し、不適各と判定した被験者についてはその理由を記載する。試験責任（分担）医師は、試験薬が処方された被験者ごとに試験薬投与の有無に関わらず、症例報告書を作成する。試験を中止した被験者においても同様に症例報告書を作成し、中止理由を記載する。

記載内容の変更・修正には、変更・修正前の記載内容が判別できるように二重線で訂正し、訂正日を記載し訂正者の捺印または署名する。

試験責任医師は作成された症例報告書の内容を確認し、記名捺印または署名し、

その写しを臨床試験部に提出する。

13-1-2 データマネジメント及び品質管理

データ登録手順及び管理方法：データ登録は症例報告書の記載内容をデータベースに入力する。データベース固定に当たり、必要なデータがすべて入力されていることを目視チェックにより確認する。その電子データをプリントアウトすることでデータ紙固定する。入力されたデータに対して品質管理を行い、不整なデータについてはカルテに戻って解決し、紙固定した紙データに修正の記録（修正日、修正者、修正理由）を残す。修正記録のある紙データの写しを臨床試験部に提出する。品質管理終了後、最終固定したデータベースをプリントアウトし、試験責任医師が署名し、その写しを臨床試験部に提出する。最終固定したデータベースは解析担当者に移管する。

13-2 解析対象集団

本試験に登録され、プロトコール治療に割り付けられた被験者のうち、下記の重大な違反があった症例を除いた被験者を対象とする。

- 同意未所得
- 試験手続き上の重大な違反
- 選択基準違反
- 除外基準違反
- 併用禁止薬
- 併用禁止療法違反

13-3. 評価項目、方法

メトホルミン投与前後で蛋白発現の差を検討。CS Analyzer による解析で比較する

免疫組織学的に陽性細胞の頻度を比較

空腹時血糖、空腹時インスリン、HOMA-R, 体重の変化を確認　耐糖能異常の変化を検討

14. 被験者の人権および安全性・不利益に対する配慮ならびに個人情報保護

14-1 人権への配慮（プライバシーの保護）

試験実施に係わる生データ類、および同意書等を扱う際は、被験者の秘密保護に十分配慮し、病院外に提出する症例報告書などでは、被験者識別コード等を使用する。試験の結果を公表する際には、被験者を特定できる情報は含まないようにする。試験の目的以外に試験で得られた被験者のデータは使用しない。

14-2 安全性、不利益への配慮

本試験中に重篤あるいは未知の副作用および随伴症状が発現した場合は、試験との関連に係わらず、速やかに厚生労働省、該当メーカーに連絡。それ以降の試験の進行については協議のうえ検討する。尚、重篤な副作用とは死亡、障害、もしくはそれにつながるおそれのある症状、事故であって、担当医が認めたものである。有害事象が発生した場合は速やかに適切な診察と処置を行う。

14-3 個人情報保護

あらかじめ被験者の同意を得ないで、同意説明文書で特定された利用目的の達成に必要な範囲を超えて、個人情報を取り扱わない。

15. 患者の費用負担

（1） 試験参加に伴って特別に費用負担が増加することはない。

（2） 原疾患、他の合併症に対する治療に準じ試験薬投与中の患者に対する検査・処置・治療は健康保険の範囲内でおこなわれる。

（3） 発生する薬剤、諸検査などの費用は健康保険制度に基づいて支払うことになる。

（4） 臨床研究のために生じる費用（保険適応外となるメトホルミン）は研究費でそれを補う。

16. 健康被害の補償および保険への加入

千葉大学医学部付属病院では、健康被害の治療費は、「自主臨床試験等における患者の費用負担の取扱要領」により取り扱う。また賠償責任に備え、試験責任医師および試験分担医師は賠償責任保険に加入する。

17. 臨床研究に関する倫理指針 およびヘルシンキ宣言への対応

本試験は臨床研究に関する倫理指針を準用するものとする。本試験に関する全ての研究者は、ヘルシンキ宣言（2000 年改訂）および臨床研究関する倫理指針に従って本試験を実施する。

18. 研究資金及び利益の衝突

本試験は奨学寄附金と科学研究費に基づいて実施され、試験責任医師及び試験分担医師により公正に行われる。本試験の計画・実施・報告において試験の結果および結果の解釈に影響を及ぼすような「起こりえる利益の衝突」は存在しないこと、また試験の実施が被験者の権利・利益を損ねることがないことを確認する。

19.研究組織

研究代表者 三橋 暁
千葉大学医学部附属病院婦人科 准教授 043-222-7171(内線 6611)

分担研究者 碓井 宏和
千葉大学医学部附属病院婦人科 助教 043-222-7171(内線 6611)

研究事務局 三橋 暁
千葉大学医学部附属病院婦人科 准教授 043-222-7171(内線 6611)

効果、安全評価委員 宇野 隆
千葉大学医学部附属病院放射線科 准教授 043-222-7171(内線 6942)

20.研究施設

千葉大学医学部附属病院婦人科

21.参考文献

1. 日本産科婦人科学会婦人科腫瘍委員会報告：2005 年度子宮体癌患者年報. 日産婦誌 59:949-979, 2007
2. Calle EE, Kaaks R. Overweight, obesity and cancer: epidemiological evidence and proposed mechanisms. Nat Rev Cancer 2004; 4: 579-91.
3. Evans JM, Donnelly LA,Emslie-Smith AM et al. Metformin and reduced risk of cancer in diabetic patients. BMJ 2005; 330:1304-5.
4. Dowling R, Zakikhani M, Fantus IG et al. Metformin inhibits mammalian target of rapamycin-dependent traslation initiation in breast cancer. Cancer Res 2007; 67:10804-12.
5. Sahra IB, Laurent K, Loubat A et al. The antidiabetic drug metformin exerts an antitumoral effect in vitro and in vivo through decrease of cyclin D1 level. Oncogene 2008; 27: 3576-86.
6. Shen ZQ, Zhu HT and Lin JF. Reverse of progestin-resistant atypical endometrial hyperplasia by metformin and oral contraceptives. Obstet Gynecol 2008; 112: 465-7.
7. Salpeter SR, Buckley NS Kahn JA, Salpeter EE. Meta-analysis: metformin treatment in persons at risk for diabetes mellitus. Am J Med 2008; 121:149-57.
