# Supplementary material for: Antitumor effects of metformin via indirect inhibition of protein phosphatase 2A in patients with endometrial cancer
Source: PLoS One. 2018 Feb 14;13(2):e0192759. doi: 10.1371/journal.pone.0192759 (PMC5812621; doi:10.1371/journal.pone.0192759)
Supplement: S3 File — (PDF) [file pone.0192759.s003.pdf]

An exploratory study on the biological effects of metformin on endometrial cancer: effect on cell cycle, MAPK, and AMPK/mTOR signaling pathway
[UMIN 000004852]

Objective

The objective of this study was to analyze the direct and indirect effects of metformin on patients with endometrial cancer at therapeutic doses.

Study objectives

Primary objectives:
To determine the effects of metformin on cell proliferation and signaling pathways, we compared the pre-treatment and post-treatment samples.
(1) Cell proliferation was evaluated via immunohistochemistry using Ki-67 antibody (Ki-67 positive cells exist in all phases of cell cycle except G0 phase) and topoisomerase II α (positive cells are expressed during S/G2/M phase)
(2) Western blot was used to analyze the differences in MAPK, AMPK, and mTOR signaling pathways and cell cycle proteins.

Secondary objectives:
(1) To determine the effect of metformin on endocrine factors (HOMA-R, insulin, glucose, leptin, and adiponectin).
(2) To determine the effect of metformin on cell proliferation in patient's serum samples obtained before and after treatment.

Eligibility criteria

Inclusion Criteria:

(1) Histologically confirmed grade 1 or 2 endometrioid adenocarcinoma
(2) Myometrium invasion ruled out via MRI
(3) Performance status = 0
(4) Serum creatinine < 0.8 mg/dl

(5) Patients who are not at risk to develop hypersensitivity with this treatment or metformin
(6) Patients must provide written informed consent to participate in the trial

Exclusion Criteria:

(1) Patients with a known history of hypersensitivity or drug allergy with metformin
(2) Patients with a history of lactic acidosis
(3) Patients on dialysis
(4) Patients with a history of shock, heart failure, myocardial infarction, and cardiovascular injury including pulmonary embolism
(5) Patients with a history of excessive alcohol use
(6) Patients with hepatic dysfunction
(7) Patients with a history of thrombosis
(8) Patients with a case of psychiatric disturbance or on antipsychotic medication
(9) Patients with diabetes mellitus
(10) Patients with a history of double cancer
(11) Patients considered unfit by the chief physician

Treatment:

Metformin (an initial dose of 750 mg/day, increased by 750 mg every week up to 1500 or 2250 mg/day) was administered to patients with endometrial cancer until the day of scheduled surgery. Endometrial tissue was obtained before and during metformin treatment via endometrial biopsy for diagnosis and hysterectomy, respectively. Patient blood samples were collected periodically for the biomarker analysis.

Sample Size:

40

Experiment:

(1) Changes in cell proliferation and cell signaling were determined in paired endometrial tissue specimens via immunohistochemistry (Ki-67 and topoisomerase IIα) and western blot (AMPK, phospho-AMPK, rpS6, phospho-rpS6, ERK1/2, phospho-ERK1/2, p27, and cyclinD1).

2) Metformin concentration was measured in surplus frozen tissue and plasma samples by using liquid chromatography-tandem mass spectrometry.

3) Changes in serum growth-stimulating potential were assessed in paired sera obtained from patients. Thymidine incorporation was measured in the presence of 2% immobilized patient serum in Ishikawa cells (an endometrial cancer-derived cell line).

4) The effect of metformin treatment on metabolism was assessed via 75-g oral glucose tolerance test before and during the treatment (approximately at week 4 of metformin treatment and a few days before surgery).

Analysis Plan:

All the data were analyzed. Mann-Whitney U test was performed to compare cell proliferation and to calculate mean, 95% confidence intervals (95% CIs), and standard deviations for continuous variables. Wilcoxon signed rank test was used to compare paired values. All comparisons were planned, and the tests were 2-sided. A p value of < 0.05 was considered statistically significant.

Funding:

The Japan Society for Promotion of Science.

Recruitment start date

January 2011

Expected end date

May 2013
