# Supplementary material for: Antitumor effects of metformin via indirect inhibition of protein phosphatase 2A in patients with endometrial cancer
Source: PLoS One. 2018 Feb 14;13(2):e0192759. doi: 10.1371/journal.pone.0192759 (PMC5812621; doi:10.1371/journal.pone.0192759)
Supplement: S2 Fig — Differences between paired specimens obtained at the time of preoperative biopsy and at surgery were evaluated using the Wilcoxon signed-rank test (Fig 3). (PDF) [file pone.0192759.s005.pdf]

```
SUMMARIZE
  /TABLES=pre post
  /FORMAT=VALIDLIST NOCASENUM TOTAL LIMIT=100
  /TITLE='Case Summaries'
  /MISSING=VARIABLE
  /CELLS=COUNT.
```

Summarize

Notes

Output Created		21-JUN-2017 21:31:28
Comments		
Input	Data	/Users/antira/Desktop/PP2A /pp2a figure/FIG 3.sav
	Active Dataset	$DataSet
	Filter	<none>
	Weight	<none>
	Split File	<none>
	N of Rows in Working Data File	6
Missing Value Handling	Definition of Missing	For each dependent variable in a table, user-defined missing values for the dependent and all grouping variables are treated as missing.
	Cases Used	Cases used for each table have no missing values in any independent variable, and not all dependent variables have missing values.
Syntax		SUMMARIZE<br>/TABLES=pre post<br>/FORMAT=VALIDLIST NOCASENUM TOTAL LIMIT=100<br>/TITLE='Case Summaries'<br>/MISSING=VARIABLE<br>/CELLS=COUNT.
Resources	Processor Time	00:00:00.01
	Elapsed Time	00:00:00.00

Case Processing Summary[a]

	Cases					
	Included		Excluded		Total	
	N	Percent	N	Percent	N	Percent
pre	6	100.0%	0	0.0%	6	100.0%
post	6	100.0%	0	0.0%	6	100.0%

a. Limited to first 100 cases.

Case Summaries[a]

		pre	post
1		1.2079	1.1749
2		3.6999	1.4717
3		2.1924	1.3310
4		3.2546	2.2230
5		2.9130	1.5236
6		1.1547	1.0849
Total	N	6	6

a. Limited to first 100 cases.

```
*Nonparametric Tests: Related Samples.
NPTESTS
  /RELATED TEST(pre post) WILCOXON
  /MISSING SCOPE=ANALYSIS USERMISSING=EXCLUDE
  /CRITERIA ALPHA=0.05  CILEVEL=95.
```

Nonparametric Tests

Notes

Output Created		21-JUN-2017 21:32:07
Comments		
Input	Data	/Users/antira/Desktop/PP2A /pp2a figure/FIG 3.sav
	Active Dataset	$DataSet
	Filter	<none>
	Weight	<none>
	Split File	<none>
	N of Rows in Working Data File	6
Syntax		NPTESTS<br>/RELATED TEST(pre post) WILCOXON<br>/MISSING SCOPE=ANALYSIS USERMISSING=EXCLUDE<br>/CRITERIA ALPHA=0.05 CILEVEL=95.
Resources	Processor Time	00:00:00.43
	Elapsed Time	00:00:00.00

Hypothesis Test Summary

	Null Hypothesis	Test	Sig.	Decision
1	The median of differences between pre and post equals 0.	Related-Samples Wilcoxon Signed Rank Test	.028	Reject the null hypothesis.

Asymptotic significances are displayed. The significance level is .05.

```
T-TEST
  /TESTVAL=0
  /MISSING=ANALYSIS
  /VARIABLES=decreaserate
  /CRITERIA=CI(.95).
```

T-Test

Notes

Output Created		21-JUN-2017 21:32:31
Comments		
Input	Data	/Users/antira/Desktop/PP2A /pp2a figure/FIG 3.sav
	Active Dataset	$DataSet
	Filter	<none>
	Weight	<none>
	Split File	<none>
	N of Rows in Working Data File	6
Missing Value Handling	Definition of Missing	User defined missing values are treated as missing.
	Cases Used	Statistics for each analysis are based on the cases with no missing or out-of-range data for any variable in the analysis.
Syntax		T-TEST<br>/TESTVAL=0<br>/MISSING=ANALYSIS<br><br>/VARIABLES=decreasera te<br>/CRITERIA=CI(.95).
Resources	Processor Time	00:00:00.01
	Elapsed Time	00:00:00.00

One-Sample Statistics

	N	Mean	Std. Deviation	Std. Error Mean
decreaserate	6	31.277000	22.9049591	9.3509104

One-Sample Test

	Test Value = 0					
					95% Confidence Interval of the Difference	
	t	df	Sig. (2-tailed)	Mean Difference	Lower	Upper
decreaserate	3.345	5	.020	31.2770000	7.239720	55.314280
