# Supplementary material for: Antitumor effects of metformin via indirect inhibition of protein phosphatase 2A in patients with endometrial cancer
Source: PLoS One. 2018 Feb 14;13(2):e0192759. doi: 10.1371/journal.pone.0192759 (PMC5812621; doi:10.1371/journal.pone.0192759)
Supplement: S6 Fig — Differences between cancer cell lines transfected with the PPP2R4 siRNA and control siRNA were evaluated using an independent t-test and the Kruskal-Wallis test (Fig 5C). (PDF) [file pone.0192759.s009.pdf]

```
DATASET ACTIVATE $DataSet.

SAVE OUTFILE='/Users/antira/Desktop/PP2A /pp2a figure/FIG 5c HEC 265 caspase.sav'
  /COMPRESSED.
GET
  FILE='/Users/antira/Desktop/PP2A /pp2a figure/FIG 5c HEC IB caspase.sav'.
DATASET NAME $DataSet WINDOW=FRONT.
```

Dataset Name

Notes

Output Created		22-JUN-2017 09:55:42
Comments		
Input	Data	/Users/antira/Desktop/PP2A /pp2a figure/FIG 5c HEC IB caspase.sav
	Filter	<none>
	Weight	<none>
	Split File	<none>
Syntax		DATASET NAME $DataSet WINDOW=FRONT.
Resources	Processor Time	00:00:00.00
	Elapsed Time	00:00:00.00

Warnings

The active dataset will replace the existing dataset named $DataSet.

```
SUMMARIZE
```

```
/TABLES=caspase BY sirna
/FORMAT=VALIDLIST NOCASENUM TOTAL LIMIT=100
/TITLE=' Case Summaries'
/MISSING=VARIABLE
/CELLS=COUNT.
```

Summarize

Notes

Output Created		22-JUN-2017 09:56:22
Comments		
Input	Data	/Users/antira/Desktop/PP2A /pp2a figure/FIG 5c HEC IB caspase.sav
	Active Dataset	$DataSet
	Filter	<none>
	Weight	<none>
	Split File	<none>
	N of Rows in Working Data File	19
Missing Value Handling	Definition of Missing	For each dependent variable in a table, user-defined missing values for the dependent and all grouping variables are treated as missing.
	Cases Used	Cases used for each table have no missing values in any independent variable, and not all dependent variables have missing values.

Notes

Syntax		SUMMARIZE<br>/TABLES=caspase BY sirna<br>/FORMAT=VALIDLIST NOCASENUM TOTAL LIMIT=100<br>/TITLE='Case Summaries'<br>/MISSING=VARIABLE<br>/CELLS=COUNT.
Resources	Processor Time	00:00:00.00
	Elapsed Time	00:00:00.00

[$DataSet] /Users/antira/Desktop/PP2A /pp2a figure/FIG 5c HEC IB caspase.sav

Case Processing Summary[a]

	Cases					
	Included		Excluded		Total	
	N	Percent	N	Percent	N	Percent
caspase * sirna	18	94.7%	1	5.3%	19	100.0%

a. Limited to first 100 cases.

Case Summaries[a]

				caspase
sirna	nontarge	1		54843.70
		2		49507.32
		3		45579.21
		4		44457.17
		5		36929.17
		6		37869.93
		7		40608.25
		8		46864.41
		9		46325.33
		Total	N	9
	pp2a	1		52346.14
		2		59843.59
		3		52448.39
		4		41858.09
		5		45472.62
		6		52486.73
		7		65665.63
		8		54038.97
		9		65850.37
		Total	N	9
	Total	N		18

a. Limited to first 100 cases.

```
T-TEST GROUPS=sirna('nontarget' 'pp2a')
  /MISSING=ANALYSIS
  /VARIABLES=caspase
  /CRITERIA=CI(.95).
```

T-Test

Notes

Output Created		22-JUN-2017 09:56:45
Comments		
Input	Data	/Users/antira/Desktop/PP2A /pp2a figure/FIG 5c HEC IB caspase.sav
	Active Dataset	$DataSet
	Filter	<none>
	Weight	<none>
	Split File	<none>
	N of Rows in Working Data File	19
Missing Value Handling	Definition of Missing	User defined missing values are treated as missing.
	Cases Used	Statistics for each analysis are based on the cases with no missing or out-of-range data for any variable in the analysis.
Syntax		T-TEST GROUPS=sirna ('nontarget' 'pp2a') /MISSING=ANALYSIS /VARIABLES=caspase /CRITERIA=CI(.95).
Resources	Processor Time	00:00:00.00
	Elapsed Time	00:00:00.00

Group Statistics

	sirna	N	Mean	Std. Deviation	Std. Error Mean
caspase	nontarge	9	44776.0541	5677.69090	1892.56363
	pp2a	9	54445.6140	8188.82525	2729.60842

Independent Samples Test

		Levene's Test for Equality of Variances		t-test for Equality of Means						
									95% Confidence Interval of the Difference	
		F	Sig.	t	df	Sig. (2-tailed)	Mean Difference	Std. Error Difference	Lower	Upper
caspase	Equal variances assumed	.977	.338	-2.911	16	.010	-9669.5599	3321.52965	-16710.888	-2628.2316
	Equal variances not assumed			-2.911	14.248	.011	-9669.5599	3321.52965	-16781.926	-2557.1936

```
*Nonparametric Tests: Independent Samples.
NPTESTS
  /INDEPENDENT TEST (caspase) GROUP (sirna)
  /MISSING SCOPE=ANALYSIS USERMISSING=EXCLUDE
  /CRITERIA ALPHA=0.05 CILEVEL=95.
```

Nonparametric Tests

Notes

Output Created		22-JUN-2017 09:57:00
Comments		
Input	Data	/Users/antira/Desktop/PP2A /pp2a figure/FIG 5c HEC IB caspase.sav
	Active Dataset	$DataSet
	Filter	<none>
	Weight	<none>
	Split File	<none>
	N of Rows in Working Data File	19
Syntax		NPTESTS /INDEPENDENT TEST (caspase) GROUP (sirna) /MISSING SCOPE=ANALYSIS USERMISSING=EXCLUDE /CRITERIA ALPHA=0.05 CILEVEL=95.
Resources	Processor Time	00:00:00.12
	Elapsed Time	00:00:00.00

Hypothesis Test Summary

	Null Hypothesis	Test	Sig.	Decision
1	The distribution of caspase is the same across categories of sirna.	Independent-Samples Kruskal-Wallis Test	.024	Reject the null hypothesis.

Asymptotic significances are displayed. The significance level is .05.
